# Supplementary material for: Effect of Enhanced Nutrition and Infection Management Intervention Packages on Antenatal Quality of Care Indicators in Rural Amhara, Ethiopia
Source: medRxiv. 2026 Jul 29:2026.07.27.26359070. Preprint. [Version 1] doi: 10.64898/2026.07.27.26359070 (PMC13618412; doi:10.64898/2026.07.27.26359070)
Supplement: Supplementary file 1 [file NIHPP2026.07.27.26359070v1-supplement-1.pdf]

medRxiv preprint doi: https://doi.org/10.64898/2026.07.27.26359070; this version posted July 29, 2026. The copyright holder for this preprint (which was not certified by peer review) is the author/funder, who has granted medRxiv a license to display the preprint in perpetuity.
It is made available under a CC-BY 4.0 International license .

Supplemental figures and tables

Table 1a: Antenatal Quality of Care (ANC QoC) components scoring

**Indicators**	**Points**
Number of ANC contacts	(1, 10)
Women whose blood pressure was measured (atleast two times) at any time during any visit	
No	0
Yes	1
Women who received infection screening - HIV, Syphilis at any visit	
No	0
Yes	1
Women whose urine sample was collected at any time during any visit	
No	0
Yes	1
Women who received tetanus toxoid vaccine (atleast two shots) at any time during any visit	
No	0
Yes	1
Women who received deworming any time during any visit	
No	0
Yes	1
Women who received Iron folic acid any time during any visit	
No	0
Yes	1

medRxiv preprint doi: https://doi.org/10.64898/2026.07.27.26359070; this version posted July 29, 2026. The copyright holder for this preprint (which was not certified by peer review) is the author/funder, who has granted medRxiv a license to display the preprint in perpetuity.
It is made available under a CC-BY 4.0 International license .

Women who received Nutritional counseling any time during any visit		
	No	0
	Yes	1
Women whose Hemoglobin was measured at any visit		
	No	0
	Yes	1
Women who received counseling on pregnancy danger signs at any visit		
	No	0
	Yes	1

Table 1b - Participant, household, and facility characteristics for ENP+EIMP vs. routine care arms.

**Characteristics**	**ENP+EIMP n(%)**	**Routine care n(%) (Neither intervention)**
Maternal age, Median (IQR)	25 (9)	26 (7)
Maternal educational status		
No formal education	297 (49.6)	283 (47.7)
Primary or lower	185 (30.9)	159 (26.8)
Secondary or higher	116 (19.4)	151 (25.4)
Maternal occupational status		
Informal	156 (26)	195 (32.9)
Agriculture/daily labor	336 (56.1)	263 (44.3)
Waged occupation	107 (17.9)	135 (22.8)
Maternal GA at study enrolment, Mean (±SD)	16.6 (±5.68)	16.5 (±5.72)
Maternal parity		
Null (0)	218 (36.2)	190 (32)
Primi (1 baby)	124 (20.6)	133 (22.4)
Multi (2-4 babies)	192 (31.8)	196 (33.1)
Grand (≥ 5 babies)	69 (11.4)	74 (12.5)
Current Pregnancy was desired, n(%)	507 (84.8)	481 (81.1)
Partner educational status		
No formal education	299 (49.9)	292 (49.2)
Primary or lower	187 (31.2)	157 (26.5)
Secondary or higher	113 (18.9)	144 (24.3)
Land Ownership, n (%)	418 (69.9)	432 (72.9)
Travel time to health facility (Minutes), Mean (±SD)	40.4 (±80.3)	42.6 (±33.3)

medRxiv preprint doi: https://doi.org/10.64898/2026.07.27.26359070; this version posted July 29, 2026. The copyright holder for this preprint (which was not certified by peer review) is the author/funder, who has granted medRxiv a license to display the preprint in perpetuity.
It is made available under a CC-BY 4.0 International license .

Abbreviations: ENP= enhanced nutrition package, EIMP= enhanced infection management package, IQR= interquartile range, SD= standard deviation, GA= gestational
